# Accurate numerical scheme for singularly perturbed parabolic delay differential equation

**DOI:** 10.1186/s13104-021-05769-4

**Published:** 2021-09-15

**Authors:** Mesfin Mekuria Woldaregay, Gemechis File Duressa

**Affiliations:** 1 Department of Applied Mathematics, Adama Science and Technology University, Adama, Ethiopia; 2 Department of Mathematics, Jimma University, Jimma, Ethiopia

**Keywords:** Boundary layer, Non-standard finite difference, Singularly perturbed, Primary 65M06, 65M12, Secondary 65M15

## Abstract

**Objectives:**

Numerical treatment of singularly perturbed parabolic delay differential equation is considered. Solution of the equation exhibits a boundary layer, which makes it difficult for numerical computation. Accurate numerical scheme is proposed using $\theta$-method in time discretization and non-standard finite difference method in space discretization.

**Result:**

Stability and uniform convergence of the proposed scheme is investigated. The scheme is uniformly convergent with linear order of convergence before Richardson extrapolation and second order convergent after Richardson extrapolation. Numerical examples are considered to validate the theoretical findings.

## Introduction

Singularly perturbed delay differential equation (SPDDE) is a differential equation in which its highest order derivative term is multiplied by small perturbation parameter and involving at least one delay term. Such type of equations have variety of applications in modelling of neuronal variability [15], in control theory [3], in description of human pupil-light reflex [14] and so on. Recently, a number of papers have been published on numerical treatment of time dependent singularly perturbed differential difference equations. In papers [1, 2, 4–8, 10–13] and [17] different authors have developed numerical scheme for treating SPDDE. The schemes in above listed papers have only linear order of convergence. In this paper, we construct second order uniformly convergent numerical scheme using non-standard FDM with Richardson extrapolation.

**Notation:** In this paper, the symbols $C, C_{1}$ and $C_{2}$ denotes a positive constant independent of the perturbation parameter and number of mesh points. The norm $\left\| .\right\|$ denotes the maximum norm.

## Considered equation

We consider a singularly perturbed parabolic delay differential equation of the form

1 $$\frac{\partial u}{\partial t}-\varepsilon \frac{\partial ^{2} u}{\partial x^{2}}+a(x)\frac{\partial u}{\partial x}(x-\delta ,t)+b(x) u(x-\delta ,t)=f(x,t),$$

on the domain $D=\Omega \times \Lambda = (0,1) \times (0,T]$ for some fixed number $T>0$ with initial and interval-boundary conditions

2 $$\begin{array}{ll} u(x,0)= u_{0}(x), x \in \bar{\Omega } , \\ u(x,t) = \phi (x,t), (x,t)\in [-\delta , 0] \times \Lambda ,\; u(1,t) = \psi (1,t), t \in \Lambda , \end{array}$$

where $0< \varepsilon \ll 1$ is singular perturbation parameter and $\delta$ is delay satisfying $\delta < \varepsilon$. The functions $a(x), b(x),f(x,t), u_{0}(x), \phi (x,t)$ and $\psi (1,t)$ are assumed to be sufficiently smooth and bounded with $b(x) \ge b^{*} > 0,$ for some constant $b^{*}$.

Solution of (1)–(2) exhibits boundary layer [4] and position of the layer depends on the conditions: If $a(x)< 0$ left layer exist. If $a(x)>0$ right layer exist.

### Some preliminary of the analytical solution

For the case $\delta <\varepsilon$, using Taylor’s series approximation for the terms containing delay $u(x-\delta ,t)$ and $u_{x}(x-\delta ,t)$ is valid [16]. Since, we assumed $\delta < \varepsilon$, we approximate (1)–(2) by

3 $$\frac{\partial u}{\partial t}-c_{\varepsilon }(x) \frac{\partial ^{2} u}{\partial x^{2}}+p(x)\frac{\partial u}{\partial x}+b(x)u(x,t)=f(x,t),$$

with initial and boundary conditions

4 $$\begin{array}{ll} u(x,0)=u_{0}(x),\; x \in {\bar{\Omega }},\\ u(0,t)=\phi (0,t),\; t\in \bar{\Lambda },\; u(1,t)=\psi (1,t),\; t\in \bar{\Lambda }, \end{array}$$

where $$c_{\varepsilon }(x)=\varepsilon -\frac{\delta ^2}{2} b(x)+\delta a(x)$$ and $$p(x)=a(x)-\delta b(x)$$.

For small values of $\delta$, (1)–(2) and (3)–(4) are asymptotically equivalent. We assume $0<c_{\varepsilon }(x) \le \varepsilon ^2-\frac{\delta ^2}{2} b^{*}+\delta a^{*} =c_\varepsilon$, where $b^{*}$ and $a^{*}$ are the lower bound for *b*(*x*) and *a*(*x*) respectively. We assume also $p(x) \ge p^{*} >0,$ implies occurrence of boundary layer near $x=1$.

#### **Lemma 2.1**

([6], Theorem 2.1) *The derivatives of the solution u(x, t) of (*3*)–(*4*) is bounded as*

5 $$\left|\frac{\partial ^{i}\partial ^{j}u(x,t)}{\partial x^{i}\partial t^{j}}\right| \le C\left (1+c_\varepsilon ^{-i}e^{-p^{*}(1-x)/c_\varepsilon }\right ),\; 0\le i\le 4, \;0\le j\le 2,$$

*where C a positive constant independent of the parameter *$c_{\varepsilon }.$

## Numerical scheme

### Temporal semi-discretization

We sub-divide the time domain [0, *T*] into *M* intervals as $t_{0}=0,\; t_{j}=j\Delta t, j=0,1,2,\ldots,M-1$, where $\Delta t= T/(M-1)$. We use $\theta$—method for semi-discretizing (3)–(4). In general, stable numerical scheme is obtained for $\frac{1}{2}\le \theta \le 1$. In case $\theta =\frac{1}{2}$ it becomes Crank Nicolson method which is second order convergent. In this discretization, for each $j=0,1,2,\ldots,M-1$ we obtain a system of BVPs

6 $$( 1+\Delta t \theta L^{\Delta t})U_{j+1}(x)=(\theta -1)\Delta tL^{\Delta t}U_{j}(x)+\Delta t[\theta f(x,t_{j+1})+(1-\theta )f(x,t_{j})],$$

where

$$L^{\Delta t}U_{j+1}(x)=- c_{\varepsilon }\frac{d^2}{dx^2}U_{j+1}(x)+p(x)\frac{d}{dx}U_{j+1}(x)+b(x)U_{j+1}(x),$$

with the boundary conditions

7 $$U_{j+1}(0)=\phi (0,t_{j+1}), \; U_{j+1}(1)=\psi (1,t_{j+1}).$$

#### **Lemma 3.1**

(Global error estimate.) *The global error estimate up to* $t_{j+1}$ *time step is given by*

8 $$\left\| E_{j+1}\right\| \le \left\{ \begin{array}{ll} C_{1}(\Delta t), \; \frac{1}{2}<\theta \le 1,\\ C_{2}(\Delta t)^{2}, \; \theta =\frac{1}{2},\end{array} \right. , \forall j=1,2,\ldots,M-1.$$

### Spatial discretization

#### Exact finite difference

To construct exact finite difference scheme, we follow the procedure in [9]. Consider the constant coefficient homogeneous differential equations of the form

9 $$-c_{\varepsilon }\frac{d^{2}u(x)}{dx^{2}}+p^{*}\frac{du(x)}{dx}+b^* u(x)=0$$

10 $$-c_{\varepsilon }\frac{d^{2}u(x)}{dx^{2}}+p^{*}\frac{du(x)}{dx}=0,$$

Equation (9) has two independent solutions namely $\exp (\lambda _{1}x)$ and $\exp (\lambda _{2}x)$ where $\lambda _{1,2}=\frac{-p^{*}\pm \sqrt{(p^{*})^2+4c_{\varepsilon }b}}{-2c_{\varepsilon }}.$

Let $x_{i}=x_{0}+ih,\; i=1,2,\ldots,N, \;\; x_{0}=0,\; x_{N}=1,\; h=\frac{1}{N}$ where *N* is the number of mesh intervals. We denote the approximate solution of *u*(*x*) at mesh point $x_{i}$ by $U_{i}$. Our main objective is to calculate a difference equation which has the same general solution as the differential equation in (9) has at the mesh point $x_{i}$ given by $U_{i}=A_{1}\exp (\lambda _{1}x_{i})+A_{2}\exp (\lambda _{2}x_{i})$. Using the theory of difference equations for second order linear difference equations in [9], we obtain

11 $$\exp \left (\frac{p^{*}h}{2c_{\varepsilon }}\right )U_{i-1}-2\cosh \left (\frac{h\sqrt{(p^{*})^2+4c_{\varepsilon }b^{*} }}{2c_{\varepsilon }}\right )U_{i}+\exp \left (-\frac{p^{*}h}{2c_{\varepsilon }}\right )U_{i+1}=0$$

is an exact difference scheme for (9). For $\varepsilon \rightarrow 0$, after arithmetic adjustment, we obtain

12 $$-c_{\varepsilon }\frac{U_{i-1}-2U_{i}+U_{i+1}}{\frac{hc_{\varepsilon }}{p^{*}}\left (\exp (\frac{p^{*}h}{c_{\varepsilon }})-1\right )}+p^{*}\frac{U_{i}-U_{i-1}}{h}=0.$$

From (12) the denominator function for second derivative discretization is $\gamma ^2 =\frac{hc_{\varepsilon }}{p^{*}}\left (\exp \left (\frac{hp^{*}}{c_\varepsilon }\right )-1\right )$. For variable coefficient equation, $\gamma ^2$ can be written as

13 $$\gamma _{i}^2 =\frac{hc_{\varepsilon }}{p(x_{i})}\left (\exp \left (\frac{hp(x_{i})}{c_\varepsilon }\right )-1\right ).$$

#### Discrete scheme

The spatial domain $\bar{\Omega }=[0,1]$, discretized with uniform mesh length $\Delta x=h$ such that $\Omega ^{N}=\{x_{i}=x_{0}+ih,\; i=1,2,\ldots,N-1, \; x_{0}=0,\; x_{N}=1,\; h=\frac{1}{N}\}$ where *N* is the number of mesh intervals. Using the discretization in (12) into the scheme in (6), we obtain

14 $$( 1+\Delta t \theta L^{h,\Delta t})U_{i,j+1}=g_{i,j+1}, \;i=1,2,\ldots,N-1,$$

where

$$L^{h,\Delta t}U_{i,j+1}=- c_{\varepsilon }\frac{U_{i-1,j+1}-2U_{i,j+1}+U_{i+1,j+1}}{\gamma _{i}^2}+p(x_{i})\frac{U_{i,j+1}-U_{i-1,j+1}}{h}+b(x_{i})U_{i,j+1}$$

and $$g_{i,j+1}=-(1-\theta )\Delta tL^{h,\Delta t}U_{i,j}+\Delta t[\theta f(x_{i},t_{j+1})+(1-\theta )f(x_{i},t_{j})]$$.

### Stability analysis and uniform convergence

We need to show that the scheme in (14) satisfies the discrete maximum principle, uniform stability estimates and uniform convergence.

#### **Lemma 3.2**

(Discrete maximum principle.) *Let* $U_{i,j+1}$ *be any mesh function satisfying* $U_{0,j+1}\ge 0,\; U_{N,j+1}\ge 0.$ *Then,* $( 1+\Delta t \theta L^{h,\Delta t})U_{i,j+1}\ge 0, i=1,2,\ldots,N-1$ *implies that* $U_{i,j+1}\ge 0, \forall i=0,1,\ldots,N.$

#### *Proof*

Suppose there exist $k\in \{0,1,\ldots,N\}$ such that $U_{k,j+1}=\min _{0\le i\le N}U_{i,j+1}<0$, which implies $k\ne 0,N$. Also we assume that $U_{k+1,j+1}-U_{k,j+1}>0$ and $U_{k,j+1}-U_{k-1,j+1}<0$. Using the assumptions made above, we obtain $( 1+\Delta t \theta L^{h,\Delta t})U_{k,j+1}< 0$, for $k=1,2,3,\ldots,N-1$. Thus the supposition $U_{i,j+1}<0$, for $i=0,1,\ldots,N$ is wrong. Hence, we obtain $U_{i,j+1}\ge 0 ,\; \forall i=0,1,\ldots,N.$ $\square$

#### **Lemma 3.3**

(Uniform stability estimate.) *Solution* $U_{i,j+1}$ *of the discrete scheme in (* 14*) satisfies the bound*

15 $$\left\| U_{i,j+1}\right\| \le \left\| g_{i,j+1}\right\| (1+\Delta t \theta b^{*})^{-1}+\max \left \{\left| \phi (0,t_{j+1})\right| , \left| \psi (1,t_{j+1})\right| \right \}.$$

#### *Proof*

Let us construct a barrier functions as $\pi ^{\pm }_{i,j+1}=\left\| g_{i,j+1}\right\| (1+\Delta t \theta b^{*})^{-1}+\max \left \{\left| \phi (0,t_{j+1})\right| , \left| \psi (1,t_{j+1})\right| \right \}\pm U_{i,j+1}$. We can easily show that $\pi ^{\pm }_{0,j+1}\ge 0$, $\pi ^{\pm }_{N,j+1}\ge 0$ and $(1+\Delta t \theta L^{h,\Delta t})\pi ^{\pm }_{i,j+1}\ge 0.$ By the discrete maximum principle, we obtain $\pi ^{\pm }_{i,j+1}\ge 0, \;\forall i=0,1,2,\ldots,N$. $\square$

Let us define the differences operators in space as $D^{+}V_{j+1}(x_{i})=\frac{V_{j+1}(x_{i+1})-V_{j+1}(x_{i})}{h}$, $D^{-}V_{j+1}(x_{i})=\frac{V_{j+1}(x_{i})-V_{j+1}(x_{i-1})}{h}$ and $D^{+}D^{-}V_{j+1}(x_{i})=\frac{(D^{+}-D^{-})V_{j+1}(x_{i})}{h}$.

#### **Theorem 3.1**

*The solution* $U_{i,j+1}$ *of (* 6 *) satisfies the truncation error bound*

16 $$\left| ( 1+\Delta t \theta L^{h,\Delta t}) \left (U_{j+1}(x_{i})-U_{i,j+1}\right ) \right| \le Ch\left [ 1+ \sup _{x_{i}\in (0,1)} \frac{\exp \left (-p^*(1-x_{i})/c_{\varepsilon }\right )}{c^3_{\varepsilon }}\right ].$$

#### *Proof*

We consider the truncation error

$$\begin{aligned} \left| ( 1+\Delta t \theta L^{h,\Delta t}) \left (U_{j+1}(x_{i})-U_{i,j+1}\right ) \right| &= \Delta t \theta\bigg \arrowvert c_{\varepsilon }\left ( \frac{d^2}{dx^2}U_{j+1}(x_{i})-\frac{D^+_{x}D^-_{x}h^2}{\gamma _{i}^2}U_{j+1}(x_{i})\right )\\ &\quad + p_{i}\left (\frac{d}{dx}U_{j+1}(x_{i})-D^-_{x}U_{j+1}(x_{i}) \right )\bigg \arrowvert \\ &\le Cc_{\varepsilon } \left| \frac{d^2}{dx^2}U_{j+1}(x_{i})-D^+_{x}D^-_{x}U_{j+1}(x_{i})\right| \\ &\quad + Cc_{\varepsilon }\left| \left ( \frac{h^2}{\gamma _{i}^{2}}-1\right )D^+_{x}D^{-}_{x}U_{j+1}(x_{i})\right| \\ & \quad+ Ch\left| \frac{d^2}{dx^2}U_{j+1}(x_{i}) \right| \\ &\le Cc_{\varepsilon } h^2\left| \frac{d^4}{dx^4}U_{j+1}(x_{i})\right| +Ch\left| \frac{d^2}{dx^2}U_{j+1}(x_{i}) \right| . \end{aligned}$$

The estimate $c_{\varepsilon }\left| \frac{h^2}{\gamma _{i}^2}-1\right| \le Ch$ used in the above expression is proved in [1]. Using bound of the derivatives of the solution in Lemma 2.1 and since $c^3_{\varepsilon }\le c^2_{\varepsilon }$, we obtain

$$\left| ( 1+\Delta t \theta L^{h,\Delta t}) \left (U_{j+1}(x_{i})-U_{i,j+1}\right ) \right| \le Ch \left [1+ \sup _{x_{i}\in (0,1)}\frac{\exp \left ( \frac{-p^*(1-x_{i})}{c_{\varepsilon }}\right )}{c^3_{\varepsilon }}\right].$$

#### **Lemma 3.4**

*For a fixed mesh N, and for* $c_{\varepsilon } \rightarrow 0,$ *we obtain*

17 $$\lim _{c_{\varepsilon }\rightarrow 0} \max _{1\le i\le N-1} \frac{\exp \left ( \frac{-p^*(1-x_{i})}{c_{\varepsilon }}\right )}{c^m_{\varepsilon }}=0,\; i=1,2,\ldots,N-1, \; m=1,2,3,\ldots$$

Using Lemma 3.4 into Theorem 3.1 gives $\left| ( 1+\Delta t \theta L^{h,\Delta t})\left ( U_{j+1}(x_{i})- U_{i,j+1}\right ) \right| \le Ch$. Applying the discrete maximum principle in Lemma 3.2, we obtain the error bound as $\left| U_{j+1}(x_{i})- U_{i,j+1} \right| \le Ch$.

#### **Theorem 3.2**

*Solution of the scheme in (* 14 *) satisfies the uniform error bound*

18 $$\sup _{0<c_{\varepsilon }\le 1} \left\| u(x_{i},t_{j+1})-U_{i,j+1}\right\| \le \left\{ \begin{array}{ll} C(N^{-1}+(\Delta t)), \; \frac{1}{2}<\theta \le 1,\\ C(N^{-1}+(\Delta t)^2), \; \theta =\frac{1}{2},\end{array} \right.$$

#### *Proof*

The uniform error bound of the scheme follows from the results of Theorem 3.1, Lemma 3.4 and the bound from temporal discretization. $\square$

### Richardson extrapolation

We apply the Richardson extrapolation technique in spatial direction to accelerate the rate of convergence of the scheme. Let $U_{i,j+1}^{2N,M}$ denoted for an approximate solution on 2*N* and *M* number of mesh points by including the mid points $x_{i+1/2}$ into the mesh points, which gives that $U^{ext}_{i,j+1}=2U_{i,j+1}^{2N,M}-U_{i,j+1}$ is an extrapolated solution. The uniform error bound becomes

19 $$\sup _{0<c_{\varepsilon }\ll 1}\left\| u(x_{i},t_{j+1})-U^{ext}_{i,j+1}\right\| \le \left\{ \begin{array}{ll} C(N^{-2}+(\Delta t)), \; \frac{1}{2}<\theta \le 1,\\ C(N^{-2}+(\Delta t)^2), \; \theta =\frac{1}{2},\end{array} \right.$$

## Numerical results and discussion

We considered two numerical examples of the form in (1)–(2) from [6, 18] to illustrate the theoretical findings of the proposed scheme.

### *Example 4.1*

$a(x)=2-x^2,\; b(x)=x^2+1+\cos (\pi x)$ and $f(x)=10t^{2}\exp (-t)(1-x)$ for $u_{0}(x)=0,\; 0\le x\le 1$ and $\phi (x,t)=0,\; x\in [-\delta , 0],\; \psi (1,t)=0$ for final time $T=1$.

### *Example 4.2*

$a(x)=2-x^2,\; b(x)=3-x$ and $f(x)=\exp (t)\sin (\pi x(1-x))$ for $u_{0}(x)=0, 0\le x\le 1$ and $\phi (x,t)=0,\;x\in [-\delta , 0],\;\; \psi (1,t)=0$ for final time $T=1$.

The exact solution of the examples are not known. We use the double mesh procedure to calculate maximum absolute error as $E^{N,M}_{\varepsilon ,\delta }=\max _{ i,j} |U^{N,M}_{i,j}-U^{2N,2M}_{i,j}|.$ The uniform error estimate is calculated using $E^{N,M}=\max _{\varepsilon ,\delta }\left| E^{N,M}_{\varepsilon ,\delta }\right|$. The uniform rate of convergence is calculated using $r^{N,M}=\log _{2}\left (E^{N,M}/E^{2N,2M}\right ).$

**Fig. 1:**
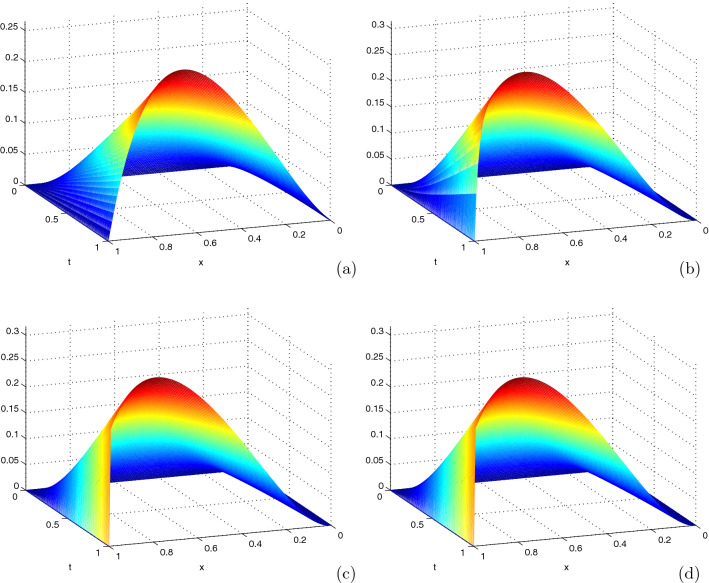
Boundary layer formation in 3D view of Example 4.2 on **a** $\varepsilon = 10^{-1}$, **b** $\varepsilon = 10^{-2}$, **c** $\varepsilon = 10^{-3}$ and **d** $\varepsilon = 10^{-4}$

Solution of Examples 4.1 and 4.2 exhibits a right boundary layer. As one observes in Fig. 1, as the perturbation parameter, $\varepsilon$ goes small; the boundary layer formation becomes more visible. In Tables 1 and 2, the maximum absolute error, the uniform error and the uniform rate of convergence of the scheme before and after Richardson extrapolation is given for different values of $\varepsilon$ and mesh numbers. As one observes the results in the tables, the maximum absolute error before Richardson extrapolation are independent of $\varepsilon$ as, the parameter $\varepsilon$ goes small. The scheme before Richardson extrapolation have linear order of convergence and the scheme after Richardson extrapolation have second order of convergence.

**Table 1:** Maximum absolute error of Example 4.1 for $\delta =0.9\varepsilon$, $\theta =\frac{1}{2}$

$\varepsilon \; \downarrow \; N=M\rightarrow$	$2^4$	$2^5$	$2^6$	$2^7$
*Before extrapolation*				
$10^{-4}$	1.4608e−02	8.1605e−03	4.3079e−03	2.2125e−03
$10^{-6}$	1.4608e−02	8.1600e−03	4.3077e−03	2.2124e−03
$10^{-8}$	1.4608e−02	8.1600e−03	4.3077e−03	2.2124e−03
$10^{-10}$	1.4608e−02	8.1600e−03	4.3077e−03	2.2124e−03
$E^{N,M}$	1.4608e−02	8.1600e−03	4.3077e−03	2.2124e−03
$r^{N,M}$	0.8401	0.9217	0.9613	-
*After extrapolation*				
$10^{-4}$	8.1605e−03	2.2125e−03	5.6425e−04	1.4182e−04
$10^{-6}$	8.1600e−03	2.2124e−03	5.6422e−04	1.4181e−04
$10^{-8}$	8.1600e−03	2.2124e−03	5.6422e−04	1.4181e−04
$10^{-10}$	8.1600e−03	2.2124e−03	5.6422e−04	1.4181e−04
$E^{N,M}$	8.1605e−03	2.2125e−03	5.6425e−04	1.4182e−04
$r^{N,M}$	1.8830	1.9713	1.9923	–

**Table 2:** Maximum absolute error of Example 4.2 for $\delta =0.9\varepsilon$, $\theta =\frac{1}{2}$

$$\varepsilon \; \downarrow$$$$N=M \rightarrow$$	$$2^4$$	$$2^5$$	$$2^6$$	$$2^7$$
*Before extrapolation*				
$10^{-4}$	9.2814e−03	6.5095e−03	3.8026e−03	2.0167e−03
$10^{-6}$	9.2806e−03	6.5094e−03	3.8028e−03	2.0170e−03
$10^{-8}$	9.2806e−03	6.5094e−03	3.8028e−03	2.0170e−03
$10^{-10}$	9.2806e−03	6.5094e−03	3.8028e−03	2.0170e−03
$E^{N,M}$	9.2814e−03	6.5095e−03	3.8028e−03	2.0170e−03
$r^{N,M}$	0.5118	0.7755	0.9149	–
*After extrapolation*				
$10^{-4}$	6.5095e−03	2.0167e−03	5.1663e−04	1.2977e−04
$10^{-6}$	6.5094e−03	2.0170e−03	5.1667e−04	1.2978e−04
$10^{-8}$	6.5094e−03	2.0170e−03	5.1667e−04	1.2978e−04
$10^{-10}$	6.5094e−03	2.0170e−03	5.1667e−04	1.2978e−04
$E^{N,M}$	7.0289e−03	2.0310e−03	5.1667e−04	1.2978e−04
$r^{N,M}$	1.7911	1.9749	1.9932	–

## Conclusion

In this paper, second order uniformly convergent numerical scheme is developed for solving singularly perturbed parabolic delay differential equation. The developed scheme is based on non standard FDM. Stability of the scheme is investigated using construction of barrier function for the solution bound. Uniform convergence of the scheme is proved. Applicability of the scheme is investigated by considering two test examples. Effects of the perturbation parameter on the solution is shown using figures and tables. The scheme is accurate, stable and uniformly convergent.

## Limitations

- The proposed scheme is not layer resolving method (i.e. there is no sufficient number of mesh points in the boundary layer region).

## Data Availability

No additional data is used for this research work.

## Abbreviations

SPDDE: Singularly perturbed delay differential equation
FDM: Finite difference method
